# Cold Atmospheric Plasma as a Novel Method for Inactivation of Potato Virus Y in Water Samples

**DOI:** 10.1007/s12560-019-09388-y

**Published:** 2019-04-29

**Authors:** Arijana Filipić, Gregor Primc, Rok Zaplotnik, Nataša Mehle, Ion Gutierrez-Aguirre, Maja Ravnikar, Miran Mozetič, Jana Žel, David Dobnik

**Affiliations:** 1 Department of Biotechnology and Systems Biology, National Institute of Biology, Večna pot 111, 1000 Ljubljana, Slovenia; 2 Jožef Stefan International Postgraduate School, Jamova cesta 39, 1000 Ljubljana, Slovenia; 3 Department of Surface Engineering and Optoelectronics, Jožef Stefan Institute, Jamova cesta 39, 1000 Ljubljana, Slovenia; 4 University of Nova Gorica, Vipavska 13, 5000 Nova Gorica, Slovenia

**Keywords:** Cold atmospheric plasma, Potato virus Y, Virus inactivation, Water decontamination

## Abstract

While one of the biggest problems we are facing today is water scarcity, enormous quantities of water are still being used in irrigation. If contaminated, this water can act as an effective pathway for the spread of disease-causing agents, like viruses. Here, we present a novel, environmentally friendly method known as cold atmospheric plasma for inactivation of viruses in water used in closed irrigation systems. We measured the plasma-mediated viral RNA degradation as well as the plasma-induced loss of viral infectivity using potato virus Y as a model virus due to its confirmed water transmissibility and economic as well as biological importance. We showed that only 1 min of plasma treatment is sufficient for successful inactivation of viruses in water samples with either high or low organic background. The plasma-mediated inactivation was efficient even at markedly higher virus concentrations than those expected in irrigation waters. Obtained results point to reactive oxygen species as the main mode of viral inactivation. Our laboratory-scale experiments confirm for the first time that plasma has an excellent potential as the eukaryotic virus inactivation tool for water sources and could thus provide a cost-effective solution for irrigation mediated plant virus transmission. The outstanding inactivation efficiency demonstrated by plasma treatments in water samples offers further expansions of its application to other water sources such as reused wastewater or contaminated drinking waters, as well as other plant, animal, and human waterborne viruses, ultimately leading to the prevention of water scarcity and numerous human, animal, and plant infections worldwide.

**Electronic supplementary material:**

The online version of this article (10.1007/s12560-019-09388-y) contains supplementary material, which is available to authorized users.

## Introduction

The availability of clean water is in continued decline due to the increasing global population and food demand, along with higher standards of living and climate change (WWAP 2018). Water scarcity has an important impact on the environment as it affects aquatic organisms, groundwater-dependent terrestrial ecosystems as well as plants and humans (Pfister et al. 2011). Over the past 4 years, water scarcity has been regarded as one of the highest global risks, in terms of its potential impact on humanity (World Economic Forum 2017). Despite this, 70% of water use worldwide goes on account of irrigation (WWAP 2018). This makes agriculture a major environmental burden in terms of water use (Ridoutt et al. 2018). To tackle this important global problem, closed irrigation systems that recycle water, such as hydroponic systems, are becoming more common. However, such systems can serve as a route for efficient and rapid transmission of pathogens in case of water source contamination. Plant pathogens can reduce seed germination, affect the yield and even destroy entire crops (Syed Ab Rahman et al. 2018).

Water-transmissible viruses are especially problematic, as they are usually resistant to wastewater treatment processes (Carducci et al. 2009) and common disinfection methods that have been developed to target mostly bacteria. Moreover, viruses can survive in water for long periods of time, can be infectious at low doses, and are the source of numerous human, animal and plant infections and epidemics (Mehle and Ravnikar 2012; Shrestha et al. 2018). Potato virus Y (PVY) is a water-transmissible plant virus that can successfully spread through irrigation systems (Mehle et al. 2014). PVY is, economically and scientifically speaking, one of the 10 most important plant viruses worldwide (Scholthof et al. 2011) and the most important potato viral pathogen which can cause up to 80% loss in crop production (Kogovšek et al. 2016). PVY isolates from the recombinant PVY^NTN^ group are the most devastating and cause mosaic, chlorotic, and necrotic lesions on leaves as well as necrotic ringspots on tubers (Kogovšek et al. 2016). High losses in potato yield pose a big problem since potato is one of the most important crops in the world (FAO 2018). In addition to potato, PVY can also infect other important crops, such as tobacco, tomato, and pepper (Scholthof et al. 2011).

Removal of viruses from irrigation systems is possible, but typically used methods can be expensive [membrane filtration, heating, ultraviolet (UV) light, ozonation], time consuming (slow filtration), require large infrastructure (slow filtration, heat), frequent maintenance (slow filtration, UV light, ozonation), produce undesirable side components (chlorination, ozonation), or need additional decontamination steps (some types of slow filtration, UV light) (Stewart-Wade 2011). The greatest weakness of all chemical processes for water decontamination is the generation of toxic by-products as well as production, transport and handling of large amounts of dangerous decontaminants. The main limitation of physical methods is that they are effective only in water areas that are in the close proximity of the operating device (Kraft 2008). Of all the disinfection methods, only thermal disinfection has been proven to be suitable for inactivation of plant viruses in hydroponic production systems (Bandte et al. 2016). Thus it is extremely important to develop and implement efficient and environmentally friendly approaches for water decontamination that do not require toxic chemicals and can be scaled up. One of the technologies that might fulfill these requirements is cold atmospheric plasma (CAP).

Plasma is the fourth state of matter and it is generated by applying energy to a gas. It is a mixture of charged particles (i.e., ions, free electrons), reactive species, UV photons, and neutral particles (i.e., molecules, atoms in the excited or ground state). Due to some of these components, with the emphasis on the reactive chemical species, CAP has great antimicrobial potential (Guo et al. 2015). The temperature of CAP at the point of application is usually < 40 °C, which makes it suitable for treating biological samples (Hoffmann et al. 2013). CAP devices for decontamination have been tested for various applications, such as in medicine and food processing, where they have been shown to be effective (Scholtz et al. 2015). They have also been used for degradation of non-biological (Bansode et al. 2017) and biological, mostly bacterial (Rashmei et al. 2016), contaminants in water. Although some studies of CAP–virus interactions have already been performed (for review see Pradeep and Chulkyoon 2016) only one brief study has examined the effects of CAP on a plant virus, tobacco mosaic virus (Hanbal et al. 2018), and only one study has described bacteriophage inactivation in water samples by CAP (Guo et al. 2018).

The aim of the present study was to evaluate the applicability of CAP for inactivation of viruses in water from closed irrigation systems. We chose PVY^NTN^ as the model virus because of its demonstrated water transmissibility and economic relevance. We showed that CAP can inactivate high concentrations of PVY^NTN^ in nutrient solution after only 1 min of treatment and suggested that the inactivation is mainly mediated by the formation of reactive oxygen species.

## Materials and Methods

**Fig. 1:**
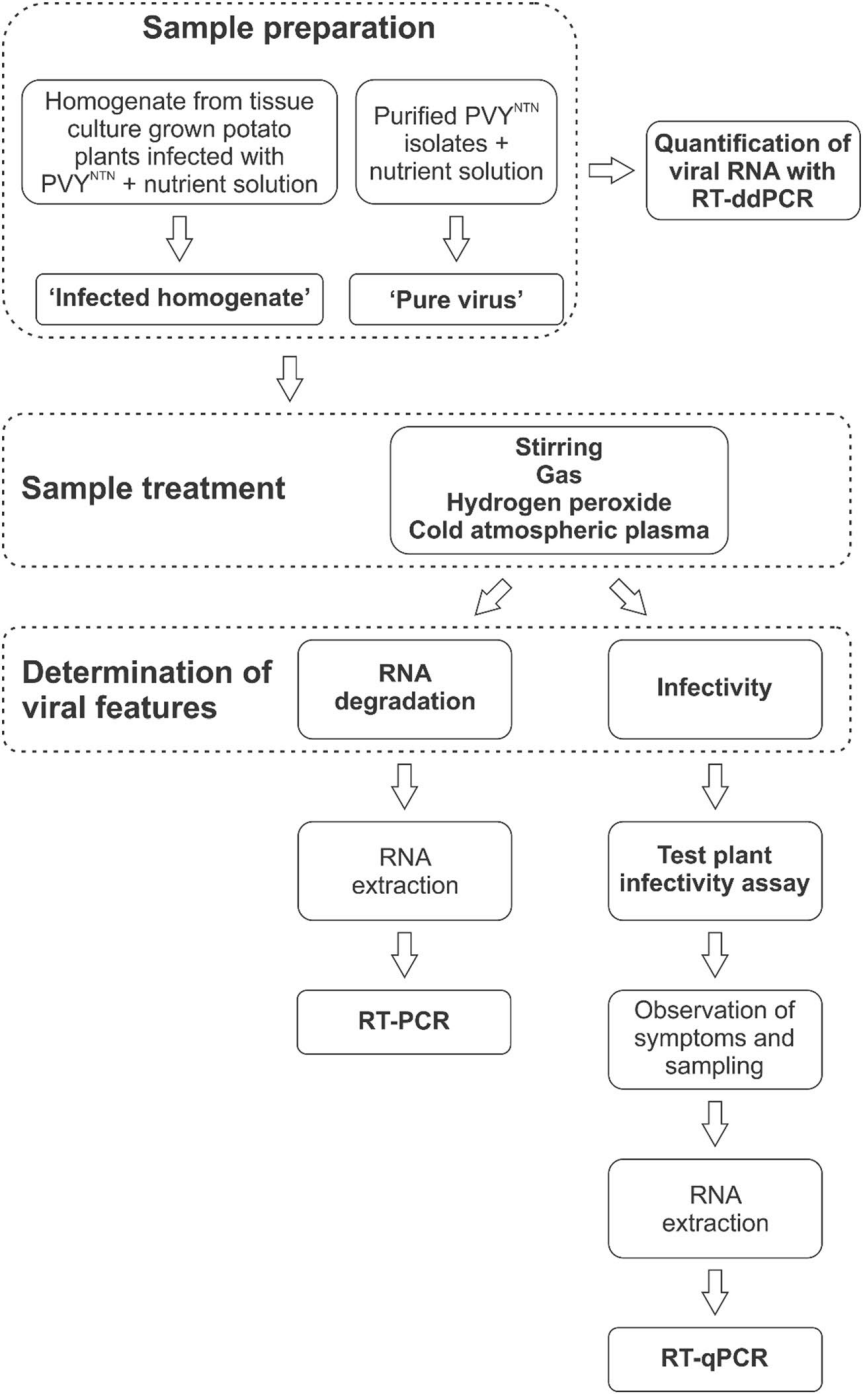
Schematic representation of the experimental design

For schematic representation of the experimental design, see Fig. 1.

### Virus Source

We used two sources of viruses in nutrient solution to create an approximation to water samples of different complexities that represent those used in closed irrigation systems. Plants infected with PVY^NTN^ were homogenized and diluted in nutrient solution to provide the complex infected water samples (henceforth referred to as ‘infected homogenate’). The less complex infected water samples contained only purified PVY^NTN^ (Online Resource 1a) diluted in nutrient solution. Ultracentrifugation-purified virus particles had lower concentration (henceforth referred to as ‘low concentration pure virus’) than chromatography-purified virus particles (henceforth referred to as ‘high concentration pure virus’). Untreated samples of infected homogenate and both types of pure virus were used as positive controls.

Each infected homogenate was prepared by grinding 88 ± 3 mg of the green parts (i.e., leaves and stems) of potato plants (*Solanum tuberosum* cv. ‘Pentland Squire’) grown in vitro cultures and infected with PVY^NTN^. This was then mixed with 20 mL nutrient solution that consisted of tap water with added minerals (Johnson et al. 1994). We divided each of the infected homogenates into two samples of 10 mL: one that served as a positive control and was not treated further, and the other that was treated with hydrogen peroxide (H_2_O_2_) (control treatment, various concentrations) or CAP (various times).

For isolation of the pure viruses, PVY^NTN^-infected tobacco and potato tissues from plants grown in the soil were prepared by grinding them in chilled (to 4 °C) grinding buffer. PVY^NTN^ was then purified using either a standard purification method that included saccharose and CsCl gradient ultracentrifugation (low concentration pure virus) or convective interaction media (CIM) monolithic chromatography (high concentration pure virus) (Rupar et al. 2013). We added the low concentration pure virus particles to 10 mL nutrient solution and then either left the sample untreated (positive controls) or treated it with simple magnetic stirring (control treatment), gas treatment (control treatment with the gas mixture used for CAP production, but in the absence of CAP), H_2_O_2_ (control treatment, various concentrations and times), or CAP (various times). The high concentration pure virus particles stayed untreated (positive control) or underwent CAP treatments (various times).

The viral RNA for all of the virus preparations was quantified prior to their treatments using reverse-transcription droplet digital PCR (RT-ddPCR). RT-ddPCR was performed with One-Step RT-ddPCR advanced kit for probes (Bio-Rad, USA) as described by Mehle et al. (2018) with minor modifications i.e., thresholds of 2400 were not always used during analyses and data with < 10,000 droplets were not discarded.

### CAP Source Characterization and Treatment

**Fig. 2:**
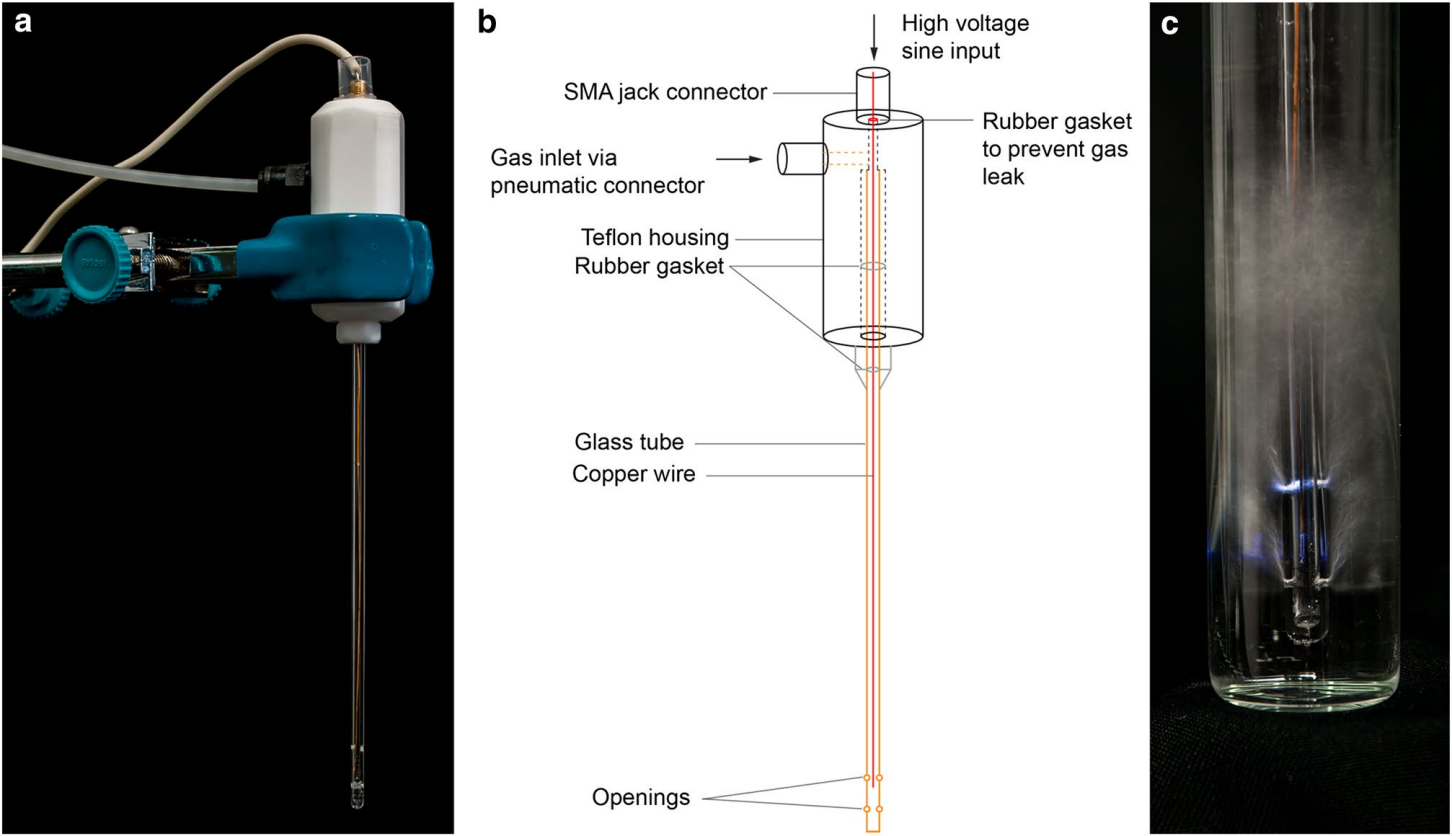
Production of cold atmospheric plasma (CAP). **a** Single electrode cold atmospheric plasma jet and **b** its schematic representation. **c** CAP treatment of a sample, during which the plasma streamers produced can be seen, as the blue-white structures in the lower part of the panel. The CAP enters the samples in the form of bubbles (blurred part of the panel) through four openings, two on each side of the glass tube (Color figure online)

We used a CAP system in the single electrode configuration to investigate the inactivation of PVY^NTN^ in the infected samples (Fig. 2). CAP was created using a mixture of argon (~ 99%) and oxygen (~ 1%), with a constant flow rate of 1 ± 0.2 L/min. The plasma or only the gas mixture was introduced into the infected samples using a perforated quartz glass tube. A copper electrode was inserted in the tube and connected to a low-frequency generator (31 kHz) that operated at a peak-to-peak voltage of 6 kV, with total average output power of ~ 3 W.

We performed treatments of the infected homogenates using CAP for 5, 15, 30, and 45 min, and 1 h in two repeats. Treatments of 2 h and 3 h were performed in a single repeat. We treated both low and high concentration pure virus using CAP for 1, 5, and 10 min in a single repeat.

**Fig. 3:**
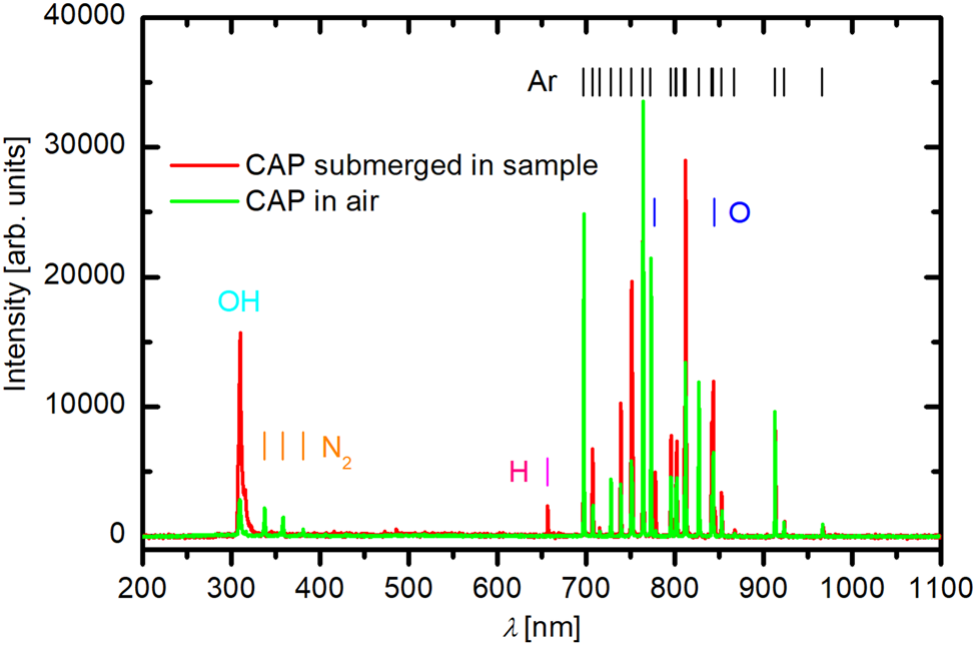
An OES spectra of a submerged CAP during treatment of low concentration pure virus and CAP in the air, in the absence of a sample. Vertical lines of the same color represent spectral print of chemical species: light blue is for OH, yellow for N_2_, pink for H, dark blue for O, and black for Ar (Color figure online)

Optical emission spectroscopy (OES) was used to observe the light emitted by the plasma during the treatments. An Avantes AvaSpec-3648 optical spectrometer with a resolution of 0.5 nm from 200 to 1100 nm was used. The integration time was set to 1 s. We measured spectra during CAP treatments of various samples: nutrient solution, low concentration pure virus, and infected homogenate. Additionally, as a control, the OES spectrum of CAP treatment without sample (CAP in the air) was recorded (Fig. 3).

### Control Treatments

We used a series of control treatments to confirm that any effect on the PVY^NTN^ arose from the CAP treatments. The first two control treatments consisted of either stirring of the low concentration pure virus on the magnetic stirrer for 1 min or treating it for 1 min with the gas mixture used for CAP production, but in the absence of CAP. The next stage control treatment included the addition of H_2_O_2_ to the samples. We applied H_2_O_2_ at final concentrations of 12.5 mg/L and 25 mg/L for 15 min with constant stirring for the infected homogenates. For the low concentration pure viruses, we applied H_2_O_2_ at final concentrations of 0.5 mg/L and 1 mg/L for 1 min and 25 mg/L for 15 min with constant stirring. Used H_2_O_2_ concentrations reflected those found to be present after various CAP treatments (see Online Resources 2 and 3).

### Reverse-Transcription PCR

To examine the effects of different treatments on degradation of viral RNA, we first extracted the PVY^NTN^ RNA from samples. RNeasy plant minikit (Qiagen, Germany) was used for RNA extractions from infected homogenates according to the manufacturer`s instructions, with minor modifications, namely, without using mercaptoethanol. RNA extractions from the pure virus were carried out with QIAmp Viral RNA minikit (Qiagen), according to the manufacturer’s instructions, with minor modifications, namely, luciferase RNA (2 ng/sample) was added to the carrier RNA prior to extraction as an external control and the final elution step was performed with 45 µL of RNAse-free water. Sterile water was used as negative control of the extraction to monitor for potential contaminations during all extractions.

After the extractions RNA was amplified for the following four PVY^NTN^ genes using reverse-transcription polymerase chain reaction (RT-PCR): P1 and P3 (which code for proteins involved in virus replication), NIa (which codes for a serine-like cysteine protease), and CP (which codes for the coat protein) (Table 1; Online Resource 1b). This selection of the viral genes enabled us to cover different parts of the viral genome. RT-PCR was prepared using One-Step RT-PCR kit (Qiagen), protocol without Q-solution, according to the manufacturer`s instructions, with minor modifications, namely, smaller volume reactions were prepared and 5 µL of template RNA was used. The cycling conditions were 30 min at 50 °C, 15 min at 95 °C, 35 cycles of 30 s at 94 °C, 1 min at 52 °C and 1 min at 72 °C, 7 min at 72 °C, and an infinite hold at 4 °C. Sterile water was used as a non-template control of RT-PCR reactions to monitor possible contaminations of the PCR reagents. We detected the amplified PCR products using agarose gel electrophoresis and considered RNA as degraded, if at least one of the four targeted genes was not amplified.

**Table 1:** Targeted genes and corresponding oligonucleotide sequences used in RT-PCR

Targets	Oligonucleotide sequences
**P1** (codes for protein involved in virus replication)	**P1_FW**: 5′-ATG GCA ACT TAC ACA TCA ACA ATC CAG-3′ **P1_R**: 5′-TTA TTG AGT AAC CTT GGA ACG TGC ATC A-3′
**P3** (codes for protein involved in virus replication)	**P3_FW**: 5′-ATG GGT ATT CCT AAT GCA TGC CCT-3′ **P3_R**: 5′-TTA CTG GTG TCG CAC ATC ATA TTC TTC C-3′
**NIa** (codes for a serine-like cysteine protease)	**NIa_FW**: 5′-ATG GCC AAA TCA CTC ATG AGA GGT TTA AG-3′ **NIa_R**: 5′-TTA TTG CTC TAC AAC AAC ATC ATG ATC AAT TAA ATC C-3′
**CP** (codes for the coat protein)	**CP_FW**: 5′-ATG GGA AAT GAC ACA ATC GAT-3′ **CP_R**: 5′-TCA CAT GTT CTT GAC TCC AA-3′

All oligonucleotides were designed within presented study. All oligonucleotides were purchased from Integrated DNA Technologies, USA

*FW* forward oligonucleotides, *R* reverse oligonucleotides

### Test Plant Infectivity Assay

We used test plant infectivity assays to examine the PVY^NTN^ infectivity in water samples after the control and CAP treatments. We mechanically inoculated two leaves of individual tobacco (*Nicotiana tabacum*, cv. ‘White Burley’) plants with nutrient solution (negative control), positive controls or treated samples. The inoculation process and growth conditions for the tobacco plants were as described in Mehle et al. (2014).

We regularly inspected test plants for development of symptoms of PVY^NTN^ infection (Online Resource 1c), and confirmed viral infectivity and systemic spreading using reverse-transcription real-time (quantitative) PCR (RT-qPCR). We sampled two developed non-inoculated upper leaves 14 ± 1 days and 32 ± 1 days post-inoculation, and pooled together all of the plant material from the plants inoculated with the same sample. We then extracted the RNA with RNeasy plant minikit according to the manufacturer`s instructions, with minor modifications i.e., without using mercaptoethanol and the final RNA elution was carried out with 150 µL of RNAse-free water. After that we performed RT-qPCR using AgPath-ID One-Step RT-qPCR mix (Ambion, USA), as described by Mehle et al. (2014) with minor modifications particularly, reactions were run in duplicates and all RNA samples were analyzed undiluted and diluted 10-fold to avoid inhibitory effects.

## Results and Discussion

The present study is the first one in the field of the eukaryotic virus inactivation by CAP for the purpose of water decontamination. Besides examining applicability of CAP for virus inactivation in contaminated water samples, we also investigated the most probable mode of viral inactivation.

Complete loss of virus infectivity (total inactivation) was achieved in 17 out of 18 CAP treatments (Table 2). Only one repeat of the infected homogenate treated by CAP for 5 min contained infective PVY^NTN^, which we detected in the upper non-inoculated leaves of the test plants. We detected PVY^NTN^ in all of the plants inoculated with the positive control samples, while we have seen no PVY^NTN^ infections for plants inoculated with the negative control (nutrient solution). Minimum time needed for inactivation of viruses in infected homogenate was 5 min, whereas only 1 min was needed for inactivation of pure virus both in high and low concentration. We speculated that difference in inactivation times originated from the amount of organic matter present in the infected homogenate, which can absorb the plasma irradiation and as such might ‘protect’ viruses from it i.e., viruses might become less accessible to the irradiation. The most obvious proof that CAP interacts with plant organic matter was a discoloration of samples in the first few minutes of treatments (Online Resource 4). Additional cause for the difference could be varied initial amount of virus in the samples, which was determined using RT-ddPCR. Concentrations of PVY^NTN^ in the infected homogenate ranged from 4.2 × 10^5^ to 6 × 10^6^ RNA copies/µL of sample, while for the low and high concentration of pure virus, average determined concentrations were 3.6 × 10^4^ and 2.7 × 10^5^ RNA copies/µL of sample, respectively (Table 2).

**Table 2:** Different treatments of water samples and their effects on the RNA and the viral infectivity

Virus sources	Treatment types	Treatment conditions (concentration and/or time)	Viral RNA concentration (copies/µL of sample)^a^	Viral RNA degradation^b^	Viral infectivity^c^
Infected homogenate	H_2_O_2_	12.5 mg/L, 15 min	4.5 × 10^5^	−	+
		25 mg/L, 15 min		−	+
	CAP	5 min^d^	7.42 × 10^5^/1.5 × 10^6^	−	+/−^e^
		15 min^d^	7.7 × 10^5^/4.4 × 10^5^	+/−^e^	−
		30 min^d^	5.6 × 10^5^/6.5 × 10^5^	+	−
		45 min^d^	4.2 × 10^5^/1.3 × 10^6^	+	−
		1 h^d^	3.6 × 10^6^/6.0 × 10^6^	−	−
		2 h	1.8 × 10^6^	−	−
		3 h	2.0 × 10^6^	+	−
Low concentration pure virus^f^	Stirring	1 min	4.0 × 10^4^	−	+
	Gas	1 min		−	+
	H_2_O_2_	0.5 mg/L, 1 min		−	+
		1.0 mg/L, 1 min		−	+
		25 mg/L, 15 min		−	−
	CAP	1 min	2.7 × 10^4^	−	−
		5 min		+	−
		10 min		+	−
High concentration pure virus^g^	CAP	1 min	2.7 × 10^5^	−	−
		5 min		−	−
		10 min		+	–

*CAP* cold atmospheric plasma treatment

^a^Viral concentration were determined in positive controls

^b^RNA was considered as degraded (+) if at least one of the four targeted genes was not amplified

^c^Viruses were considered infective (+) if we detected them with RT-qPCR in upper, non-inoculated leaves of test plants 2 and/or 4 weeks after the inoculation

^d^Two repeats of CAP treatments were performed

^e^One repeat positive (+), other repeat negative (−)

^f^PVY^NTN^ purified from infected tobacco or potato tissue using a classic purification method that included saccharose and CsCl gradient ultracentrifugation

^g^PVY^NTN^ purified from infected tobacco or potato tissue using CIM monolithic chromatography

Treated samples were tested for the presence of intact viral RNA by monitoring four targeted genes. We showed that the PVY^NTN^ RNA was successfully degraded by the CAP treatments after 15 min for the infected homogenate, and after 5 min for low concentration pure virus (Table 2). RNA was not degraded in any of the positive controls. Since RNA was not degraded in all of the experiments in which infectivity was abolished, it is likely that CAP also damages viral coat proteins, which destabilizes the virus particles. Indeed, coat protein damage alone might be enough to inactivate viruses. This is supported by the findings of different research groups which showed that coat protein damage after CAP treatment was a main mode of inactivation of different bacteriophages and mammalian viruses: bacteriophage lambda and MS2, human adenovirus and feline calicivirus (Aboubakr et al. 2018; Wu et al. 2015; Yasuda et al. 2010; Zimmermann et al. 2011).

To confirm that the viral inactivation was due to CAP treatment, we used control treatments that included H_2_O_2_, gas, or stirring. None of them had any effects on the PVY^NTN^ RNA degradation, for either the infected homogenate or the pure virus (Table 2). Moreover, in the infectivity assays, across all of the control treatments, only the highest H_2_O_2_ treatment (25 mg/L) of the pure virus for 15 min effectively reduced the PVY^NTN^ infectivity. However, the same treatment did not affect the PVY^NTN^ infectivity in the infected homogenate (Table 2). This can be explained by either higher availability of the organic material (including viruses) in the infected homogenate with which the H_2_O_2_ can interact or by the presence of the plant enzymes in the infected homogenate that can degrade the H_2_O_2_ (Zámocký et al. 2012). The data for H_2_O_2_ as a control treatment confirm its implication in plasma-mediated virus inactivation. However, the greater PVY^NTN^ inactivation obtained with CAP, compared to H_2_O_2_ alone, suggests that other plasma components are also involved in this CAP-mediated PVY^NTN^ inactivation.

We confirmed this with the OES measurements (Fig. 3) where we observed increased concentration of OH and O species that probably served as the precursors for production of different reactive oxygen species. Ar, O, and H atoms emission lines and OH emission system (A^2^Σ^+^–X^2^Π) were observed for all CAP treatments (Online Resource 5). The presence of OH emission system and Balmer H emission line in the OES spectra proves that water vapor is dissociated in the plasma. The intensities of spectral features did not change during the treatments, regardless of the sample type. A new spectral feature, N_2_ emission bands, can be seen only in the OES spectrum of CAP in the air. These are present due to the diffusion of the ambient air into the plasma stream. The OH is also present in the free air CAP because of the humidity in the ambient air. However, the OH intensity is much smaller compared to the submerged CAP, thus indicating that the water from the samples is evaporated and dissociated in the plasma. We did not detect any response in the range between 200 and 300 nm, the wavelengths at which UV radiation damages nucleotides in different ways (USEPA 2006). That is why we concluded that UV radiation could not have any impact on the virus inactivation. This leaves reactive oxygen species as the crucial CAP components of viral inactivation, an argument supported by various research groups (reviewed in Guo et al. 2015).

We have performed here a pioneering study using CAP treatment for eukaryotic virus inactivation in water samples. The use of CAP in our experiments effectively inactivated PVY^NTN^ in water samples, both in combination with the high organic background from the plant debris (infected homogenate) and in the pure PVY^NTN^ form. The inactivation was efficient even though the PVY^NTN^ genome concentrations were significantly higher than those expected in irrigation waters (Mehle et al. 2014). These new findings confirm the potential that CAP treatments hold in the field of virus inactivation in irrigation water. They also lay the groundwork to further studies on other waterborne viruses of plant, animal and human origins, and on the opportunities for the scaling up of these CAP treatments. Plasma systems might also prove useful for decontamination of other water sources, such as wastewater, drinking water, and water for recreational use. Implementation of plasma systems in wastewater treatment plants would significantly reduce their running and maintenance costs, and the space required (Barillas 2015). That might prove to be an excellent alternative for many countries that reuse wastewater for irrigation without prior disinfection due to economic limitations and the scarcity of fresh water supplies (Moazeni et al. 2017). Implementation of plasma systems might thus have important positive effects on water quality and might provide solutions that are greatly needed today. To make this implementation as smooth as possible, additional studies are required to define the exact mechanism(s) of action and whether CAP treated water can have any effects on human, animal, and plant cells.

## Electronic supplementary material

Below is the link to the electronic supplementary material.

Supplementary material 1 (DOCX 3249 kb)

Supplementary material 2 (AVI 1331 kb)
