# Supplementary material for: Cold Atmospheric Plasma as a Novel Method for Inactivation of Potato Virus Y in Water Samples
Source: Food Environ Virol. 2019 Apr 29;11(3):220–8. doi: 10.1007/s12560-019-09388-y (PMC6689025; doi:10.1007/s12560-019-09388-y)
Supplement: Supplementary file 1 — Supplementary material 1 (DOCX 3249 kb) [file 12560_2019_9388_MOESM1_ESM.docx]

**Supplementary Material**

**Cold Atmospheric Plasma as a Novel Method for Inactivation of Potato Virus Y in Water Samples**

Arijana Filipić^1,2^, Gregor Primc^3^, Rok Zaplotnik^3^, Nataša Mehle^1^, Ion Gutierrez-Aguirre^1^, Maja Ravnikar^1,4,^ Miran Mozetič^3^, Jana Žel^1^, David Dobnik^1^

^1^ National Institute of Biology, Department of Biotechnology and Systems Biology, Večna pot 111, 1000 Ljubljana, Slovenia

^2^Jožef Stefan International Postgraduate School, Jamova cesta 39, 1000 Ljubljana, Slovenia

^3^Jožef Stefan Institute, Department of Surface Engineering and Optoelectronics, Jamova cesta 39, 1000 Ljubljana, Slovenia

^4^University of Nova Gorica, Vipavska 13, 5000 Nova Gorica, Slovenia

*****Corresponding author: Arijana Filipić, [arijana.filipic@nib.si](mailto:arijana.filipic@nib.si)

Journal: [Food and Environmental Virology](https://link.springer.com/journal/12560)

**
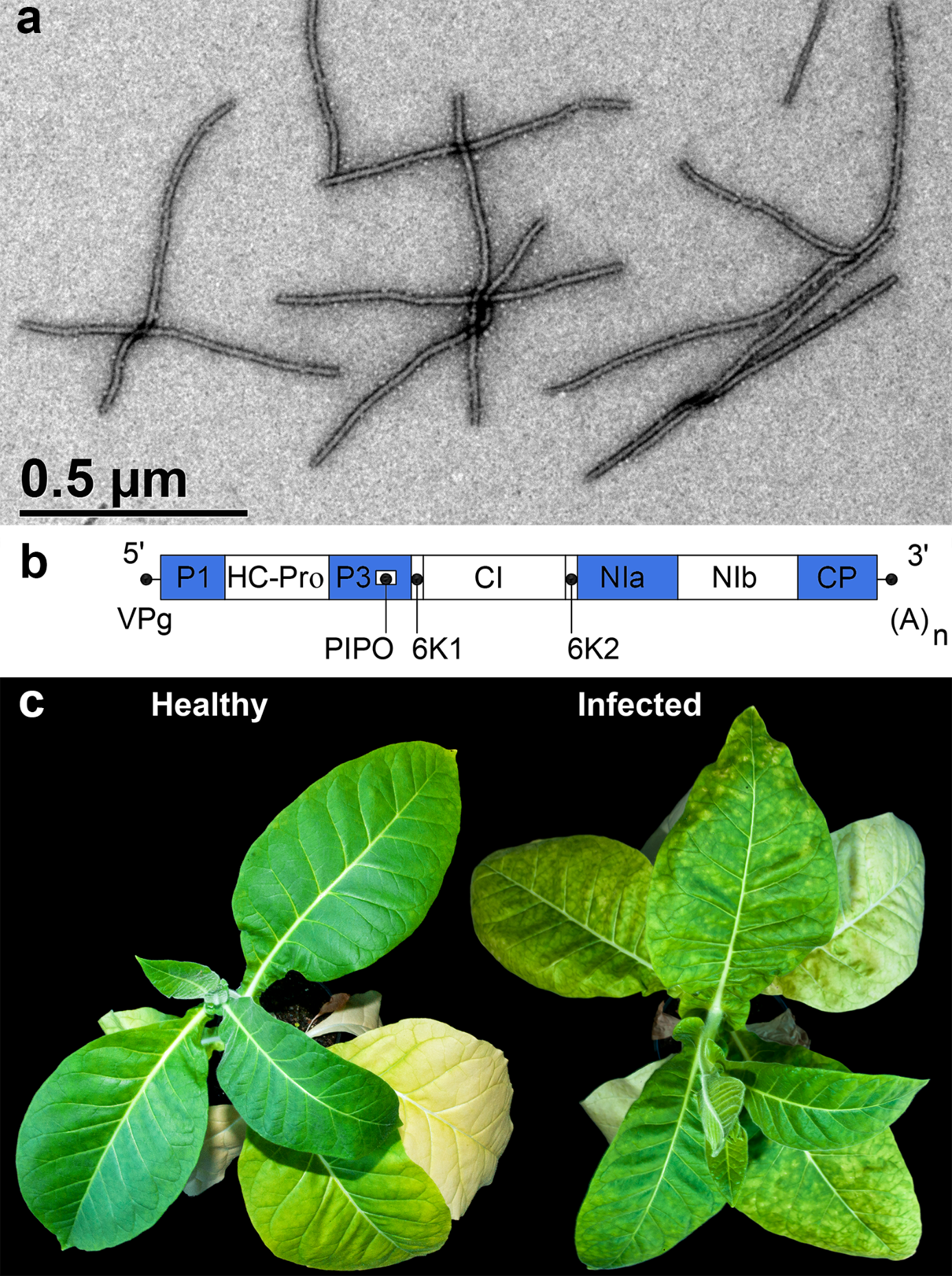
**

**Online Resource 1.** a. Representative transmission electron microscopy micrograph of PVY^NTN^ (photo by Magda Tušek Žnidarič). b. Schematic representation of the PVY^NTN^ genome. The four genes along the genome analyzed using RT-PCR are marked in blue: P1, P3, Nia, and CP. c. Representative healthy (left) and PVY^NTN^-infected (right) tobacco plants.

**Online Resource 2.** Measurements of hydrogen peroxide

Concentration of H_2_O_2_ was measured in every sample before and after the treatment for all treatment types. For that purpose, semi-quantitative Quantofix Peroxid 25 test strips (Macherey-Nagel, Germany) were used. Concentrations measured prior to any treatment were always 0 mg/L of H_2_O_2_. Results of the measurements are presented in the Online Resource 3.

**Online Resource 3.** Results of semi-quantitative H_2_O_2_ measurements directly after treatments

| Virus source | Treatment type | Treatment  conditions  (concentration and/or time) | H_2_O_2_ concentration (mg/L)^a^ |
| --- | --- | --- | --- |
| Infected homogenate | H_2_O_2_ | 12.5 mg/L; 15 min | 0 |
|  |  | 25 mg/L; 15 min | 0 |
|  | CAP | 5 min^b^ | 2-5/2-5 |
|  |  | 15 min^b^ | 25/10-25 |
|  |  | 30 min^b^ | 25/25 |
|  |  | 45 min^b^ | 25/25 |
|  |  | 1 h^b^ | 25/25 |
|  |  | 2 h | 25 |
|  |  | 3 h | 25 |
| Low concentration | Stirring | 1 min | 0 |
| pure virus^c^ | Gas | 1 min | 0 |
|  | H_2_O_2_ | 0.5 mg/L; 1 min | 0.5 |
|  |  | 1.0 mg/L; 1 min | 1 |
|  |  | 25 mg/L; 15 min | 25 |
|  | CAP | 1 min | 1 |
|  |  | 5 min | 2-5 |
|  |  | 10 min | 10 |
| High concentration | CAP | 1 min | 0,5 |
|  |  | 5 min | 2 |
| pure virus^d^ |  | 10 min | 5-10 |

CAP, Cold atmospheric plasma treatment

^a^, The highest possible measured concentration was 25 mg/L

^b^, Two repeats of CAP treatments were performed

^c^, PVY^NTN^ purified from infected tobacco or potato tissue using a classic purification method that included saccharose and CsCl gradient ultracentrifugation

^d^, PVY^NTN^ purified from infected tobacco or potato tissue using CIM monolithic chromatography

**Online Resource 4.** Discoloration of plant homogenate, prepared as described in the Manuscript, section 2.1. Virus source, after 7 min of CAP treatment (see Supplementary material 2).

**
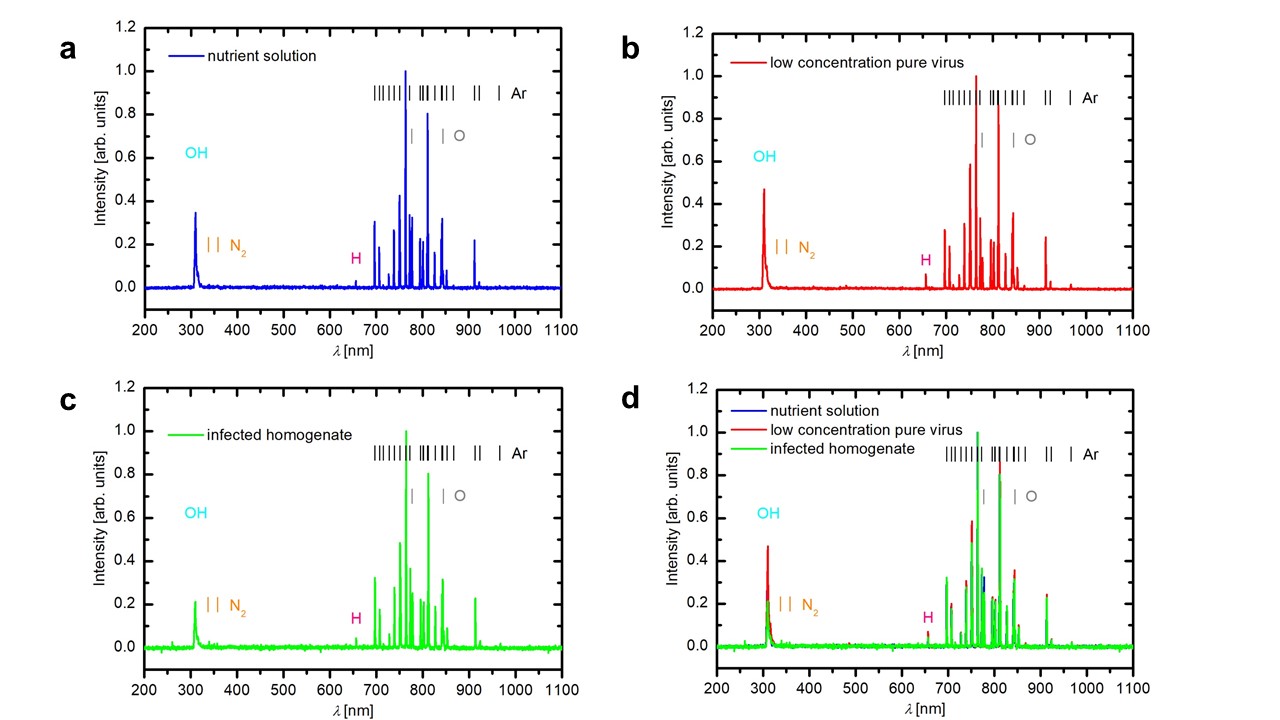
**

**Online Resource 5.** Normalized optical emission spectra of the CAP jet during treatment of various samples: a. nutrient solution, b. low concentration pure virus, c. infected homogenate and d. all treatments together. All the spectral features are the same for all the samples, only their intensities vary due to different transmittance of liquid samples.
